# High spatial resolution dataset of La Mobilière insurance customers

**DOI:** 10.1038/s41597-022-01174-z

**Published:** 2022-03-11

**Authors:** Alice Battiston, Emanuele Massaro, Claudia R. Binder, Rossano Schifanella

**Affiliations:** 1 University of Turin, Via Giuseppe Verdi, 8, 10124 Turin, TO Italy; 2 Present Address: European Commission Joint Research Center, Via E. Fermi, 2749, 21027 Ispra, VA Italy; 3 HERUS Lab, EPFL, Bˆatiment GR Station 2 CH – 1015, Lausanne, Switzerland; 4 ISI Foundation, Via Chisola 5, 10126 Turin, TO Italy

**Keywords:** Interdisciplinary studies, Geography

## Abstract

We present the La Mobilière insurance customers dataset: a 12-year-long longitudinal collection of data on policies of customers of the Swiss insurance company La Mobilière. To preserve the privacy of La Mobilière customers, we propose the data aggregated at two geographical levels, based on the place of residence of the customer: postal areas and municipalities. For each geographical area, the data provides summary statistics on: (i) the demographic characteristics of the customer base, (ii) characteristics of vehicles insurance policies and (iii) characteristics of housing and building insurance policies. To assess the validity of the data, we investigate the temporal consistency of the data and the representativeness of La Mobilière customer base along several dimensions (total population, percentage of foreigners, etc.). We also show how the insurance data can reliably model the spatial patterns of socio-economic indicators at a high geographical resolution. We believe that the reuse of this data provides an opportunity for researchers to broaden the socio-economic characterization of Swiss areas beyond the use of official data sources.

## Abstract (summary)

**Table:** 

Measurement(s)	private property insurance
Technology Type(s)	digital curation
Factor Type(s)	year • zip code • municipality
Sample Characteristic - Location	Switzerland

## Background & Summary

La Mobilière is an all-sector Swiss insurance group operating exclusively in Switzerland and Liechtenstein, with different brands according to the language of the different regions: La Mobilière (French), Die Mobiliar (German) and La Mobiliare (Italian). Founded in 1826, it is the oldest private insurance company in Switzerland and it is the market leader for personal property insurance, with a market share of more than 29%. On January 1, 2018, La Mobilière reached the threshold of two million customers, representing more than 20% of the Swiss population at the time. In this paper, we present aggregated and privacy-preserving data views that combine information from customers that have insured vehicles or buildings with the company in the 12-year period between 2008 and 2019. To preserve the anonymity of the policyholders, the data are aggregated at two geographical levels, based on the place of residence of the customers: postal areas (ZIP codes) and municipalities. The former level enables analyses at a highly granular spatial resolution, while the latter allows the linkage of the insurance data with other data sources typically collected at this spatial level (e.g., census).

The fine-grained geographical information included in this dataset is a key to link specific individual characteristics at a city level to any attribute that can be measured at the level of statistical census areas. Indeed, countries, regions and cities are increasingly favouring alternative means of gathering information, instead of the *traditional* techniques of sending out printed forms, interviewing people in person, or via the use of online questionnaires. Alternatively, they are looking to indirect means of collecting data, taking advantage of a wide spectrum of administrative data streams that act as a proxy for the variables of interest. In this direction, customer insurance records represent a valuable input to model the socio-economic substrate of cities, and an opportunity for policy makers and researchers to broaden the scope of their studies. Social scientists raised the issue of representativeness and sampling bias of large scale digital data. For example, Sivarajah *et al*.^1^ have shown how age, gender, ethnicity, socio-economic status, online experiences, and Internet skills, influence the social network sites that users generally adopt. This has implications for the extent of the conclusions that a study could claim given a particular audience. Insurance customers records share with census data the similar size, reliability, and structural complexity^2^. However, they differ in their spatio-temporal granularity and collection costs. In fact, the information of insurance customers is collected constantly by the provider while the census runs generally with a multi-year frequency due to its organizational costs. A downside is the proprietary nature of insurance records that could invalidate the possible benefits for a broader community. However, we embrace the vision of initiatives like *Data Collaboratives* (https://datacollaboratives.org) that proposes a new form of collaboration, beyond the public-private partnership model, in which participants from different sectors, in particular companies, exchange their data to create public value.

In the research literature, insurance data have been mostly used to study the impact of specific diseases^3,4^, to propose models of customers fraud detection^5,6^, to explore the correlation between census-based socio-economic indicators and injury causes^7^ or to evaluate disparities within health care systems^8^. Different non-traditional datasets have been used to characterize the socio-economic footprint of urban systems and municipalities^9–14^. Examples from the literature also indicate that housing and vehicles insurance data can act as proxy measures for crime risk^15^ and road safety^16^ respectively. Along this line, this dataset provides an opportunity for researchers to broaden the characterization of the socio-economic substrate of Swiss areas, including information from insurance logs not otherwise available in the official data. The availability of granular spatial data at the level of postal codes also allows to model geographical patterns at a high-resolution not otherwise observable in the official data.

## Methods

**Fig. 1:**
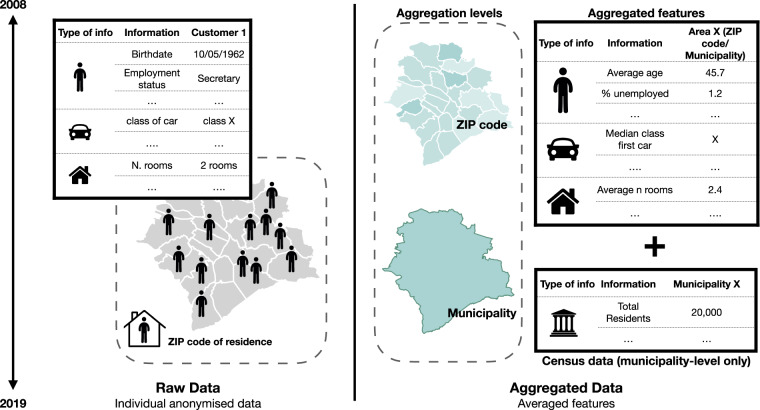
The La Mobilière insurance customers dataset aggregation process. From the company, we obtained different information of anonymized customers by their place of residence at ZIP-code level. We aggregated the raw individual-level information by extracting different indicators for each ZIP code and municipality. The dataset at municipality level was additionally enriched with census data. In the Figure, we show an example of four indicators extracted from the raw dataset.

In this section, we outline how the data was collected and aggregated. An high-level sketch of the process is provided in Fig. 1.

### Insurance data

The data described in this paper was provided by the insurance company La Mobilière, the Swiss market leader for personal property insurance. The raw data contains information on housing and vehicles insurance policies of anonymized customers over a 12-year period spanning between 2008 and 2019. Each data record refers to a single insurance policy for a vehicle, a building or both. While we could generally expect a one-to-one mapping between policies and customers, there may be cases where the same customer owns multiple policies. As an example, consider a two-member household composed of a couple (wife and husband), who owns two cars and their family house. Furthermore, the wife runs a commercial activity and owns a small shop. In this situation, the couple may be registered in the La Mobilière-System as three customers with the following policies: (1) car-insurance policy of the wife, (2) car-insurance policy of the husband and insurance policy for the family house, (3) housing-insurance for the shop. As unique customers cannot be identified in the data, we will assume that each policy corresponds to a new customer. For each policy, three sets of variables are available in the raw data: (i) *demographic information on the policyholder*, such as age, gender, ZIP code of the residential area, employment and civil status of the customer; (ii) *information on vehicles insurance policies*, such as the Canton where the car is registered, the brand and the price of the vehicle, as well as the record of the claims and the respective compensations; (iii) *information on housing insurance policies*, such as the ZIP code of the insured house, the standard of furniture, the number of rooms, the price of the building and the logs of the claims. To preserve anonymity of the customer base of La Mobilière, we release geographically aggregated data and use two aggregation levels: municipalities and postal areas (ZIP-code areas). The aggregation levels and the aggregation process are described in the following paragraphs.

### Geographic areas and census statistics

We aggregated the raw data at two geographical levels, based on the place of residence of the policyholder: *postal code (ZIP-code) areas* and *municipalities*, as defined from the Swiss Federal Statistical Office (https://www.bfs.admin.ch/). The choice of providing data at the level of municipalities as a complement to the postal code aggregation is motivated by the fact that this spatial level is commonly used by the Swiss Federal Statistical Office. Therefore, official socio-economic indicators that can be used to complement or cross-validate our dataset are mostly available at this aggregation level. It is noteworthy that the geographical boundaries of both Swiss ZIP-codes and municipalities were periodically redefined over the past decades, mostly leading to the aggregation of small municipalities into larger ones. To ensure the mapping between ZIP-codes and municipalities is correctly specified at all points in time, we provide the data at municipality-level only for those 2,095 municipalities whose administrative boundaries have not changed over the 12-year period for which we have data. These correspond to 95.1% of all the 2,202 Swiss municipalities (2020 definition) and 95.0% (8.1 million out of 8.5 million) of the resident population (2018 population data).

### Features extraction and geographic aggregation

From the initial set of variables available in the raw dataset, we performed a feature engineering step and we selected the variables of interest with the aid of a domain expert. As a result of this process, we ended up with three classes of geographically-aggregated features, outlined below. When applicable, for each feature we computed the mean, the standard deviation, the 95-confidence interval for the mean and the value of the 5th, 25th, 50th, 75th and 95th percentile. In the following section, the generic word *area* is used to indicate both municipalities and postal areas. If the data is available at one geographical level only, this is explicitly stated in the wording.

#### Demographic features

The first group of variables encodes demographic information about the customer base of La Mobilière within each area. In particular, we provide information about: the number of customers in the area, their age, the fraction of customers who is property owner, the fraction of non-Swiss customers, the average number of children aged 0–26 for customers with at least one child in the age-group and the fraction of female customers. To investigate the representativeness of La Mobilière data along several dimensions, in the technical validation we show how these characteristics correlate with analogous information from the census.

#### Vehicles insurance-related features

The second set of variables relates to vehicles insurance policies. We extracted information on:

- average characteristics of insured vehicles, e.g. their class (e.g. SUV, MLK, etc. A full description of the classes of cars is provided in Table 1), their price, the year of matriculation and their cylinder capacity (hereinafter CCM).

**Table 1:** Classes of cars insured by customers of La Mobilière.

Class of car	Description
MKL	Middle class
KWA	Small car
VAN	Compact Van/minivan
OMK	Upper middle class
UMK	Lower middle class
MIC	Micro class
CPE	Coupé/sports car
SUV	Sports utility vehicles/off-road vehicles
LKL	Luxury class
CAB	Cabriolet/Roadster
ATV	All-terrain vehicle/quad
SMA	Street motorcycle
ROL	Scooter
CHO	Chopper motorcycle/cruiser motorcycle
GMA	Off-road motorcycle
- average characteristics of vehicles insurance policies, e.g. the number of claims over the last 5 years, their (cumulative) value and the premium class.

The same policy/customer number may contain information about more than one vehicle. The features provided in the geographically-aggregated dataset refer to the main vehicle insured under each policy/customer number only.

#### Housing insurance-related features

The third set of variables relates to housing and building insurance policies. As for the previous set of variables, we extracted features on:

- average characteristics of houses and buildings insured by La Mobilière customers, e.g. the standard of furniture (from 0 to 4), the number of rooms, the year of construction and the type of building (e.g. a detached house for residential use, condominium for residential use, commercial building, etc. A full description of the types of buildings is provided in Table 2).

**Table 2:** Types of buildings insured by customers of La Mobilière.

Type of building (German)	Description	Code
Einfamilienhaus	Detached house	DH
Wohn- und Geschäftsgebäude	Residential and commercial buildings	RCB
Mehrfamilienhaus bis 3 Wohnungen	Multi-family house with up to 3 apartments	M3less
Mehrfamilienhaus über 3 Wohnungen	Apartment building with 3 apartments	M3
Eigentumswohnung	Condominium	Cond
Heim, Spital, Anstalt	Home, hospital, institution	HHI
Landwirtschaftliches Gebäude	Agricultural building	AB
Spezialgebäude	Special building	SB
Geschäftsgebäude	Commercial building	CB
Parkhaus, Einstellhalle	Parking garage	P
Schule, Bildungsgebäude	School, educational building	School
Vereins-, Sport- und Freizeithaus	Club, sports and leisure center	Sport
Schloss	Manor House	Manor
Gebäude der öffentlichen Hand	Public buildings	Public
Kirche, Kloster	Religious building	RB
- average characteristics of housing insurance policies, e.g. the number of claims over the last 5 years, their (cumulative) value and the premium class.

## Data Records

We made the dataset aggregated at municipality and zip-code levels available through Figshare^17^ under the Creative Commons International license 4.0 (CC BY 4.0). We confirm that we have appropriate approval to share this data. The raw individual-level anonymized data can be requested to La Mobilière (https://www.mobiliere.ch/) for properly motivated and framed research purposes. For both geographical levels and for each *year*, the data can be downloaded as *.csv* files (ZIP_ *year*.csv & municipality_combinedData_ *year*.csv). As census information is only available for the period 2010–2018, the municipality-level datasets are provided for these years only and include 7 additional fields extracted from census data. Below we provide a list of all the fields included in each dataset.

- **unemp**. Proportion of customers living in the area who report to be unemployed. *Data type*: Float.
- **age_mean**. Average age of the customers living in the area. *Data type*: Float.
- **frac_own**. Fraction of customers living in the area who is house owner. *Data type*: Float.
- **frac_foreign**. Fraction of non-Swiss customers living in the area. *Data type*: Float.
- **child_mean**. Average number of children aged 0–26 of customers with at least one children in the age group and living in the area. *Data type*: Float.
- **custom**. Number of customers living in the area. Please consider the clarification in Section **Insurance data**. *Data type*: Integer.
- **frac_women**. Proportion of female customers living in the area. *Data type*: Float.
- **car1_custom_frac**. Proportion of customers living in the area who insured one or more cars. *Data type*: Float.
- **car1_***class***_frac**. Proportion of cars insured by customers living in the area belonging to class *class*. A list of classes of cars is provided in Table 1. *Data type*: Float.
- **car1_pr_mean**. Average price of cars insured by customers living in the area (in CHF). *Data type*: Float.
- **car1_y_pct50**. Median year of matriculation of cars insured by customers living in the area. *Data type*: Float.
- **car1_ccm_mean**. Average CCM of cars insured by customers living in the area. *Data type*: Float.
- **car1_claim_mean**. Average number of claims over the last 5 years on cars insured by customers living in the area. *Data type*: Float.
- **car1_sumcl_mean**. Average sum of claims over the last 5 years on cars insured by customers living in the area (in CHF). *Data type*: Float.
- **car1_prem_mean**. Average premium class of cars insured by customers living in the area. *Data type*: Float.
- **build_custom_frac**. Proportion of customers living in the area who insured one or more buildings. *Data type*: Float.
- **cl_furn_pct50**. Median class of furniture of buildings insured by customers living in the area. The class of furniture ranges between 0 and 4. *Data type*: Float.
- **rooms_mean**. Average number of rooms of buildings insured by customers living in the area. *Data type*: Float.
- **build_ins_mean**. Average total sum of insured values of the building insured by customers living in the area (in CHF). *Data type*: Float.
- **build_y_pct50** Median year of construction of building insured by customers living in the area. *Data type*: Float.
- **build_***type***_frac**. Proportion of building insured by customers living in the area belonging to building type *type*. A list of types of buildings is provided in Table 2. *Data type*: Float.
- **build_claim_mean**. Average number of claims over the last 5 years related to houses and buildings insured by customers living in the area. *Data type*: Float.
- **build_sumcl_mean**. Average sum of claims over the last 5 years related to houses and buildings insured by customers living in the area (in CHF). *Data type*: Float.
- **build_prem_mean**. Average class of premium of houses and building insured by customers living in the area. *Data type*: Float.
- **BFS**. Municipality numeric identifier (available in the municipality-level datasets only). *Data type*: Numeric code.
- **municipality**. Municipality name (available in the municipality-level datasets only). *Data type*: String.
- **ZIP**. ZIP code identifying the postal code area (available in the ZIP-code level datasets only). *Data type*: Numeric code.

Where applicable, all averaged measures are accompanied with their standard deviation (fields with sufx _std), the 95% confidence interval for the mean (sufx _ci95), and the values of the 5th, 25th, 50th, 75th, and 95th percentiles (sufx _pct*value*). When the desired quantile lies between two data points, we adopted a linear interpolation method to compute the measure. The datasets at municipality-level provide seven additional fields obtained from the census:

- **pop_census**. Total residents in the municipality. *Data type*: Integer.
- **age_0_19_census**. Proportion of residents aged [0–19] in the municipality. *Data type*: Float.
- **age_20_64_census**. Proportion of residents aged [20–64] in the municipality. *Data type*: Float.
- **age_65+_census**. Proportion of residents aged 65 or above in the municipality. *Data type*: Float.
- **pop_d_census**. Population density. *Data type*: Float.
- **frac_foreign_census**. Proportion of non-Swiss residents in the municipality. *Data type*: Float.

Insurance and census data provided in the same dataset refer to the same year.

## Technical Validation

Being the market leader for personal property insurance, data from La Mobilière are well-suited and reliable when it comes to describing characteristics of the insurance products, such as the average characteristics of the policies (e.g. premium classes) and of insured properties (e.g. CCM of the cars or number of rooms per buildings). The data also provides a reliable picture of behavioural features linked to the insurance policies, e.g. geographical patterns in the number of claims and their values. Furthermore, a recent contribution^18^ has shown that La Mobilière data can successfully be used to predict geographical patterns exhibited by Swiss municipalities along several socio-economic dimensions (demographic, economic, transportation, housing, space and territory), using geographical regression models techniques. On one side, this analysis demonstrated the ecological validity of this dataset - by linking observables in the insurance data to expected outcomes among the population (e.g. percentage of customers who insured a car and fraction of commuters using public transportation), on the other side it showed the potential of insurance customers data to characterize socio-economic processes embedded in space. Yet, the data still requires a technical validation with respect to the following elements:

- the internal consistency of the data, to ensure the data display similar patterns over the 12 years. The presence of abrupt deviations may indeed suggest changes in the data collection process over time, potentially invalidating the longitudinal dimension of the dataset.
- the geographic and temporal variation in the representativeness of La Mobilière customers with respect to the Swiss population, along three dimensions: total population, proportion of foreign residents and the age profile of resident population. This component of the validation is carried out at municipality level, for which data from the census are available.

### Internal consistency

**Fig. 2:**
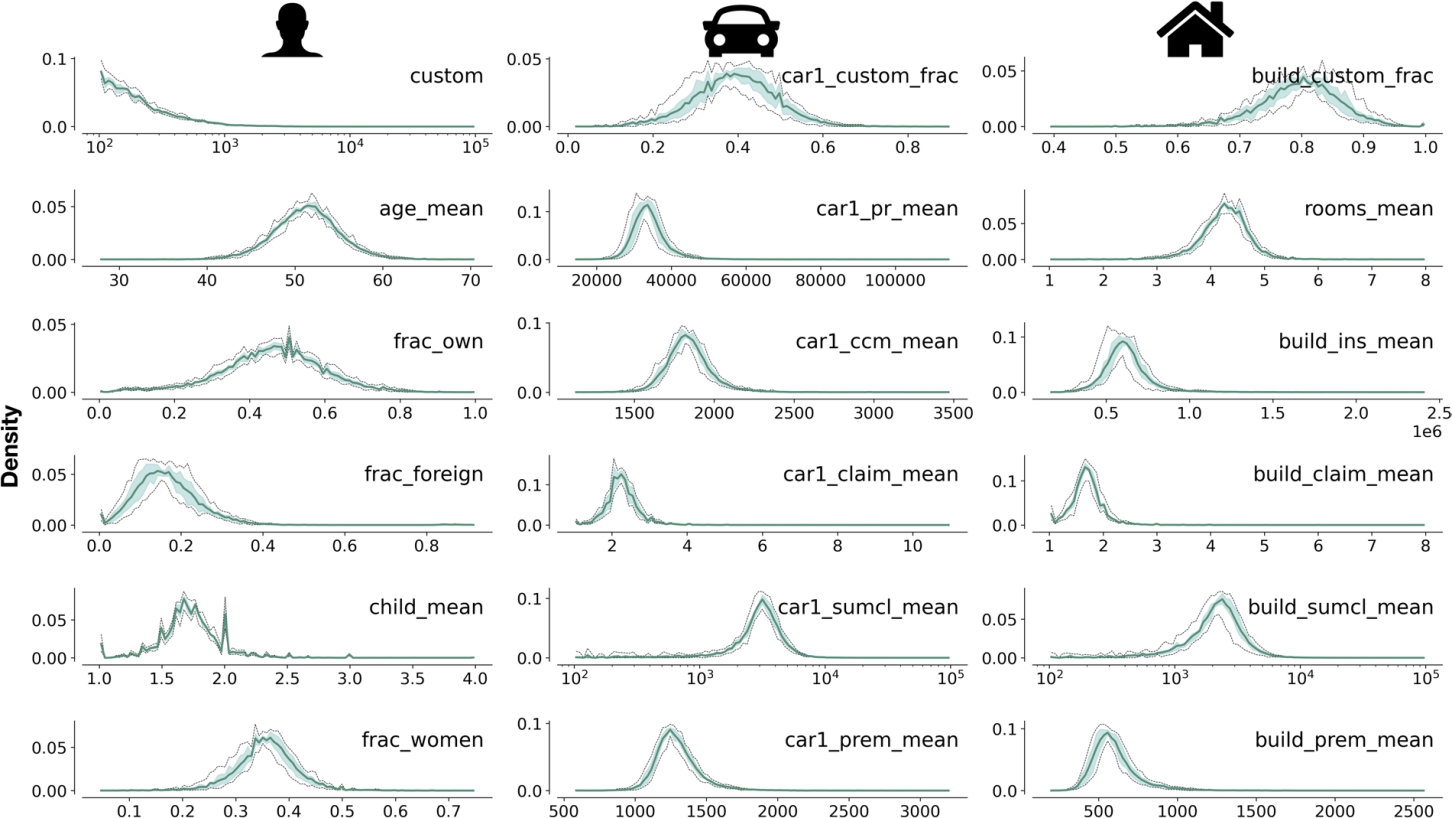
Average densities of selected variables. The solid line represents the average of the densities of the selected feature measured for each ZIP code over the 12-year period spanning between 2008 and 2019. The shaded area and the dotted black line depict the interquartile range and the minimum and maximum of the yearly densities respectively.

**Fig. 3:**
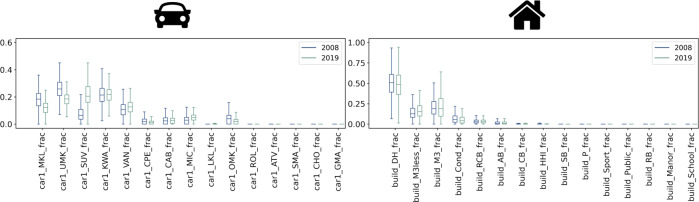
Box plots of the proportion of insured cars per class and insured buildings per type, 2008 and 2019. The charts show the box plots of the proportion of insured cars for the 15 classes of cars (left) and insured buildings for the 15 types of buildings (right) measured at ZIP-code level, for 2008 and 2019.

To investigate the internal consistency of La Mobilière data, we look at changes in the distribution of the extracted features over time. To this scope, Fig. 2 depicts the average (solid line) and interquartile range (shaded area) of the yearly distributions of selected average features, aggregated at ZIP-code level. Moreover, Fig. 3 displays the box plots of variables related to the proportion of cars per class and the proportion of buildings per type in 2008 and 2019, again measured at ZIP code level. Overall, the interquartile range in Fig. 2 displays little variation from the average density. This suggests that the data are highly-consistent over time and do not display abrupt changes.

### Representativeness

In this section, we compare La Mobilière data aggregated at municipality-level with corresponding information from the census in order to study the temporal and geographical heterogeneity in the representativeness of La Mobilière data along three dimensions: total population, proportion of foreigners and residents in three age groups: 0–19, 20–64 and 65+.

#### Population

**Fig. 4:**
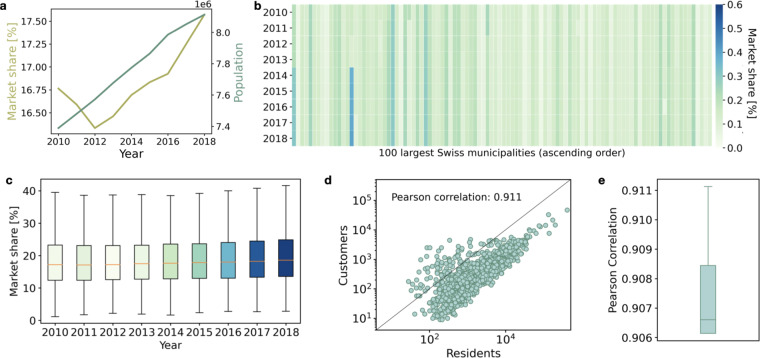
Representativeness of insurance data in terms of population. (**a**) shows the increase in the Swiss population and the market-share of La Mobilière between 2010 and 2018 at nation-level. The figures are obtained averaging over the 2,095 municipalities whose administrative boundaries have not changed in the last decade. (**b**) depicts the market share of the 100 largest Swiss municipalities (according to 2018 population data), in ascending order (the largest municipality being placed on the right). Each column represents a municipality and each row one year). (**c**) provides the box plots for the market shares measured at municipality level by year. (**d**) is a scatter plot of residents vs La Mobilière customers in 2018. Each point represents a municipality. (**e**) shows the distribution of the yearly correlations between residents and La Mobilière customers. A market share greater than 1 can result from the ambiguity in the counting of unique customers described in Section Methods.

We use a measure of market share to assess the temporal and geographical variation in the penetration of La Mobilière data (Fig. 4). For each municipality *i*, we define the market share as:

1 $$m{s}_{i}=\frac{{C}_{i}}{{N}_{i}}$$

where *C*_*i*_ is the number of La Mobilière customers in municipality *i* and *N*_*i*_ is the total number of residents in the same area (obtained from the census). The nation-level market share for each year is constructed by averaging over the municipalities. At national level, after an initial drop, we observe a progressive though limited increase in the penetration (from about 16.4% to around 17.6%) of La Mobilière between 2012 and 2018 (Fig. 4(a)). In the last three years, the rise in the market share has accelerated, suggesting that the customer base of La Mobilière has grown faster than the overall population. Importantly, we observe only limited temporal and geographical variation in the market share across municipalities (Fig. 4(b,c)). Along the temporal dimension, Fig. 4(b) shows the variation in the market share over the 10-year period of the 100 largest Swiss municipalities. For the vast majority of these large municipalities, the market share stayed constant over time. Along the geographical side, Fig. 4(c) shows the boxplots of the market shares at municipality level for each year. With almost 50% of the distribution lying within less than 10 percentage points in each year, this indicates limited geographical variation. As such, the data on the number of customers mimic well the geographical distribution of the population. This result is confirmed in Fig. 4(d,e), where we show the high correlation between the number of customers and the number of residents. In particular, the Pearson’s correlation coefficient * ρ* stands at 0.911 in 2018 (d) and ranges between 0.906 and 0.911 for earlier years (e). It should be noted that a market share greater than 1 for some small municipalities can results from the ambiguity in the counting of unique customers described in the previous section.

#### Foreigners

**Fig. 5:**
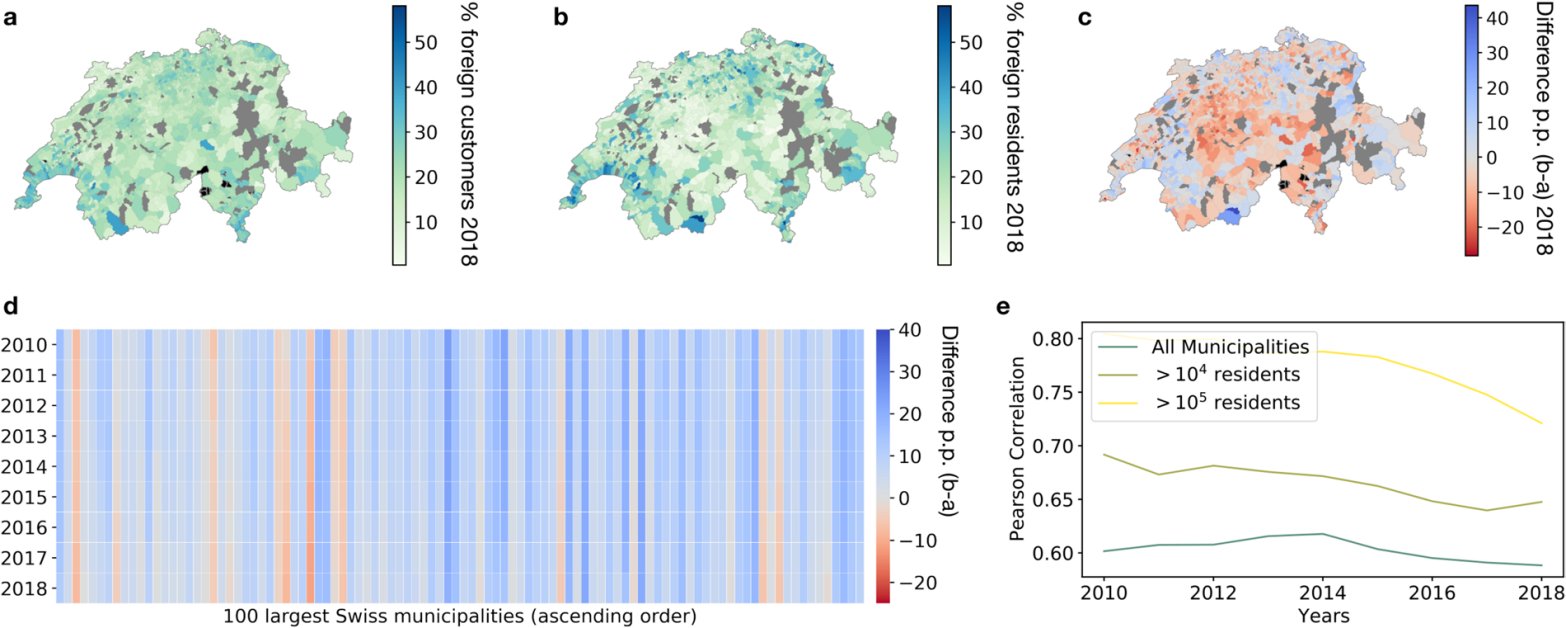
Representativeness of insurance data in terms of percentage of foreigners. Panels (**a**) and (**b**) depict the proportion of foreign customers in 2018 La Mobilière data and foreign residents from the census. Grey areas indicate municipalities excluded from the dataset due to changes in the administrative boundaries over time. Black areas in (**a**) indicate municipalities with no customers. (**c**) depicts the difference among the percentage of foreigners from the two datasets (census data- La Mobilière data). Grey areas indicate municipalities excluded from the dataset due to changes in the administrative boundaries over time. Black areas indicate municipalities with no customers. (**d**) depicts the difference in percentage points between census data and La Mobilière data for the 100 largest Swiss municipalities (according to 2018 population data), in ascending order (the largest municipality being placed on the right). Each column represents a municipality and each row one year of data. (**e**) shows the correlation between the percentage of La Mobilière’s foreign customers and the percentage of Swiss residents by year and municipality size.

The second dimension along which we explore the representativeness of La Mobilière data concerns the percentage of foreigners. Figure 5(a,b) presents data on the percentage of foreign customers (a) and foreign residents (b) for 2018. Figure 5(c) displays the difference in percentage points between the two measures (*residents*–*customers*). The same information measured over the 10-year period for the 100 largest Swiss municipalities is provided in Fig. 5(d). Large municipalities typically have a larger proportion of foreigners than what observed in La Mobilière data, with the difference being stable over time. Despite differences in actual values, we still observe a positive correlation between La Mobilière customers and the Swiss census in terms of percentage of foreigners, with a *ρ* ∼ 0.6 for all years (Fig. 5(e)). The correlation is stronger in relatively larger cities compared to smaller ones, suggesting that the insurance data may mimic better relatively more populated areas than less populated areas.

#### Age

**Fig. 6:**
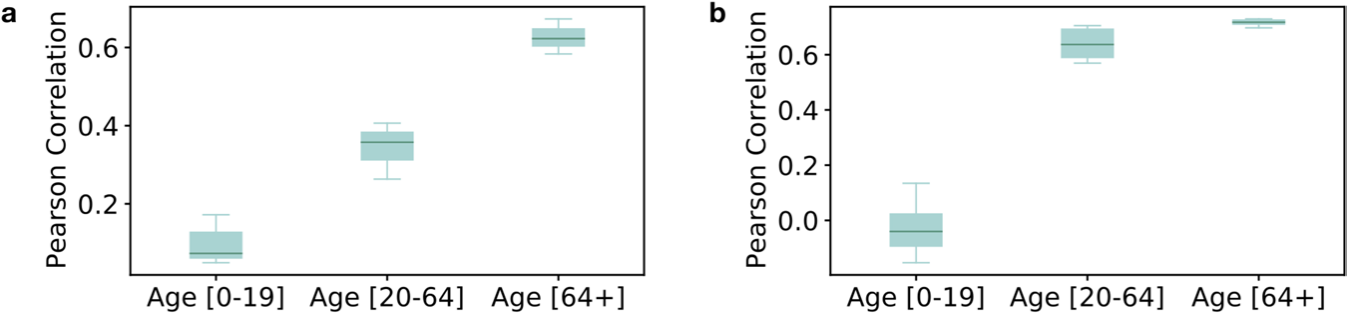
Representativeness of insurance data along the age profile. (**a**) presents the distribution of correlations between the age of customers of La Mobilière and Swiss residents (measured on all municipalities with customers) for the period 2010–2018. (**b**) presents the same information for municipalities with 10,000 or more inhabitants.

The latter dimension considered in the technical validation relates to the age distribution. In particular, we explore the correlation between the share of customers in each age group ([0–19], [20–64], [64+]) and the share of the population in the same age group (Fig. 6(a)). Once again, the comparison is performed at municipality level. We observe a positive correlation for the age-group 20–64 and 64+ (*ρ* > 0.4 and *ρ* > 0.6 respectively). By contrast, the correlation disappears (*ρ* ∼ 0.09) for customers aged 0–19. This behavior is expected since children and teenagers are not usually the owners of insurance policies on vehicles or houses. If we consider the biggest municipalities with more than 10’000 residents only, we observe stronger correlations for people in the two older age groups, while it remains similarly low for children and teenagers (Fig. 6(b)).

### Insurance data as proxy for socio-economic indicators at a high geographical resolution

**Fig. 7:**
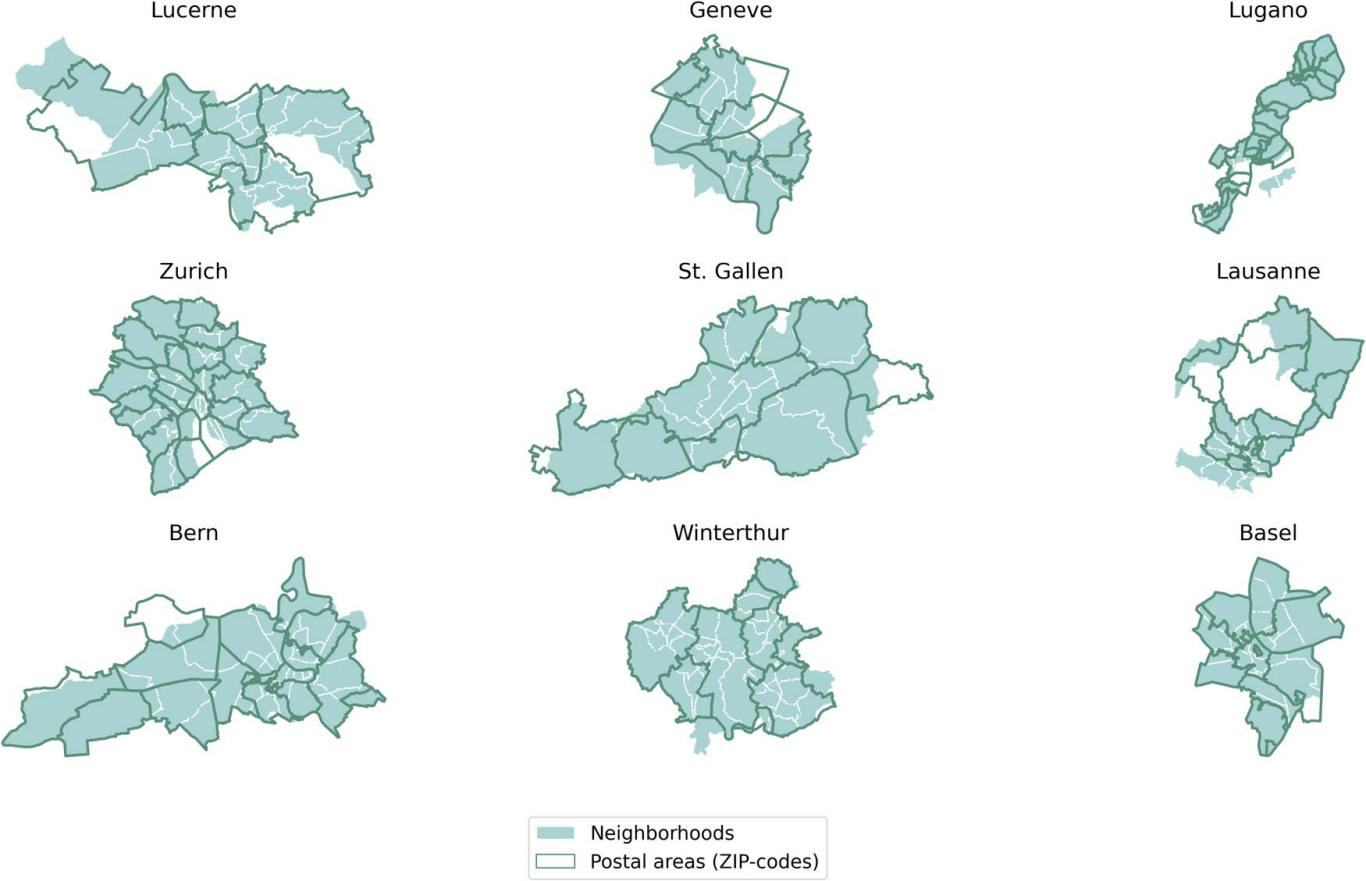
Postal areas and neighborhoods for nine Swiss municipalities. The charts depict the rough correspondence between postal code areas and neighborhoods for the nine Swiss municipalities of Lucerne, Geneve, Lugano, Zurich, St. Gallen, Lausanne, Bern, Winterthur and Basel.

To the best of our knowledge, data collections at ZIP-code level are not publicly available for the Swiss territory. This strongly undermines our ability to validate the dataset at this spatial level, but also indicates the potentiality for the insurance data to act as proxies for socio-economic characteristics not otherwise available at this very granular geographical level. In this section, we explore this possibility and rely on ground-truth data extracted from the data collection on City Statistics - Quality of life in cities^19^. This data collection provides an atlas of demographic and socio-economic indicators measured at neighborhood-level^20^ for the nine Swiss municipalities of Basel, Zurich, St. Gallen, Bern, Lucerne, Winterthur, Geneve, Lausanne and Lugano. Of particular interest for our analysis are the statistics on the proportion of foreign residents, the median age of residents and the number of detached houses the 100 apartments, which all have a direct counterpart in the insurance data. Ground-truth measures at ZIP-code level are generated exploiting the rough correspondence between postal areas and neighborhoods (Fig. 7). In particular, for each ZIP code within the nine municipalities covered by the atlas, we construct ground-truth measures by taking the weighted average of the 2019 statistics measured at neighborhoods-level. For each ZIP code, we define the weights to be proportional to the spatial intersection of the ZIP code itself with each of the neighborhoods. This means that the set of weights for the ZIP code *j* are given by ${w}_{j,i}=\frac{area\_intersectio{n}_{j,i}}{area\_dis{s}_{j}}$ where *area*_*intersection* is the area of the spatial intersection between the ZIP code *j* and the neighborhoods *i* and *area*_*diss*_*j*_ is the total area of the ZIP code covered by the neighborhoods obtained by spatial dissolving *area*_*intersection*_*j,i*_ over *i*. By construction, ${\sum }_{i}{w}_{j,i}=1$. To make sure we do restrict the analysis to ZIP codes with sufficient coverage by the available neighborhoods, we only consider those ZIP codes for which the coverage (*area*_*diss*_*j*_) is a least 30% of the overall area of the ZIP code.

**Fig. 8:**
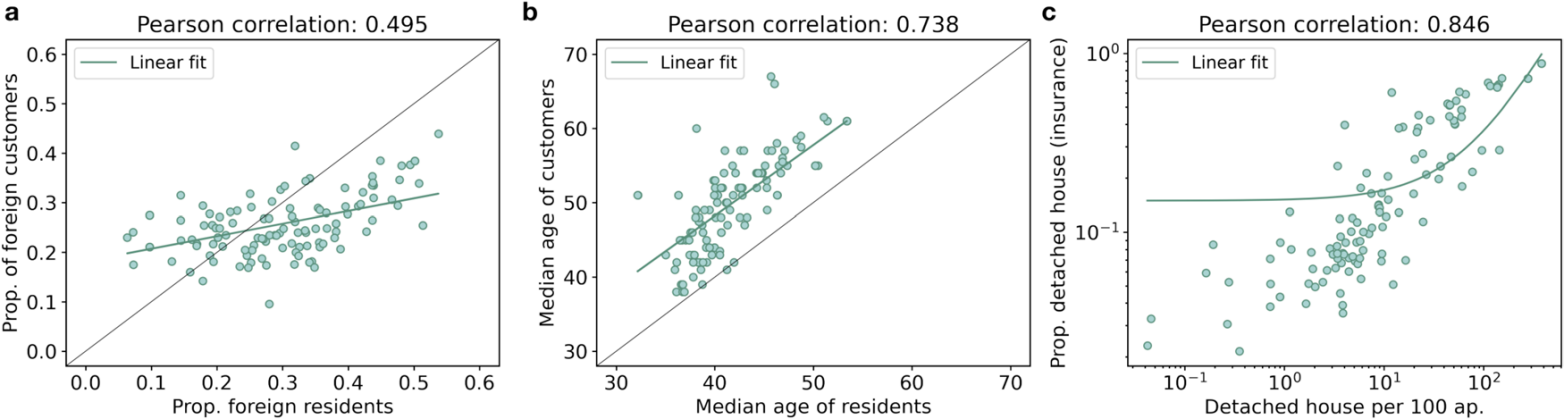
Technical validation, zip-code level dataset. (**a**) is a scatter plot of the proportion of foreign residents vs the proportion of foreign customers, measured at ZIP-code level. (**b**) is a scatter plot of the median age of residents vs the median age of La Mobilière customers, measured at ZIP-code level. (**c**) is a scatter plot of the number of detached houses per 100 apartments vs the proportion of detached houses among insured buildings (log-log scale), measured at ZIP-code level.

As for data at municipality-level, the positive correlations between insurance data and ground-truth population-level indicators confirm the high-representativeness of the insurance data along the available dimensions, also at this granular spatial resolution. In particular, the insurance data appears to mimic well the geographical distribution of population data along the age profile (*ρ* ~ 0.74), while the correlation is weaker for the proportion of foreigners (*ρ* ~ 0.50), as displayed in Fig. 8(a,b). A correlation of 0.85 is observed between the proportion of detached houses among insured buildings and the number of detached houses per 100 apartments (Fig. 8(c)).

**Fig. 9:**
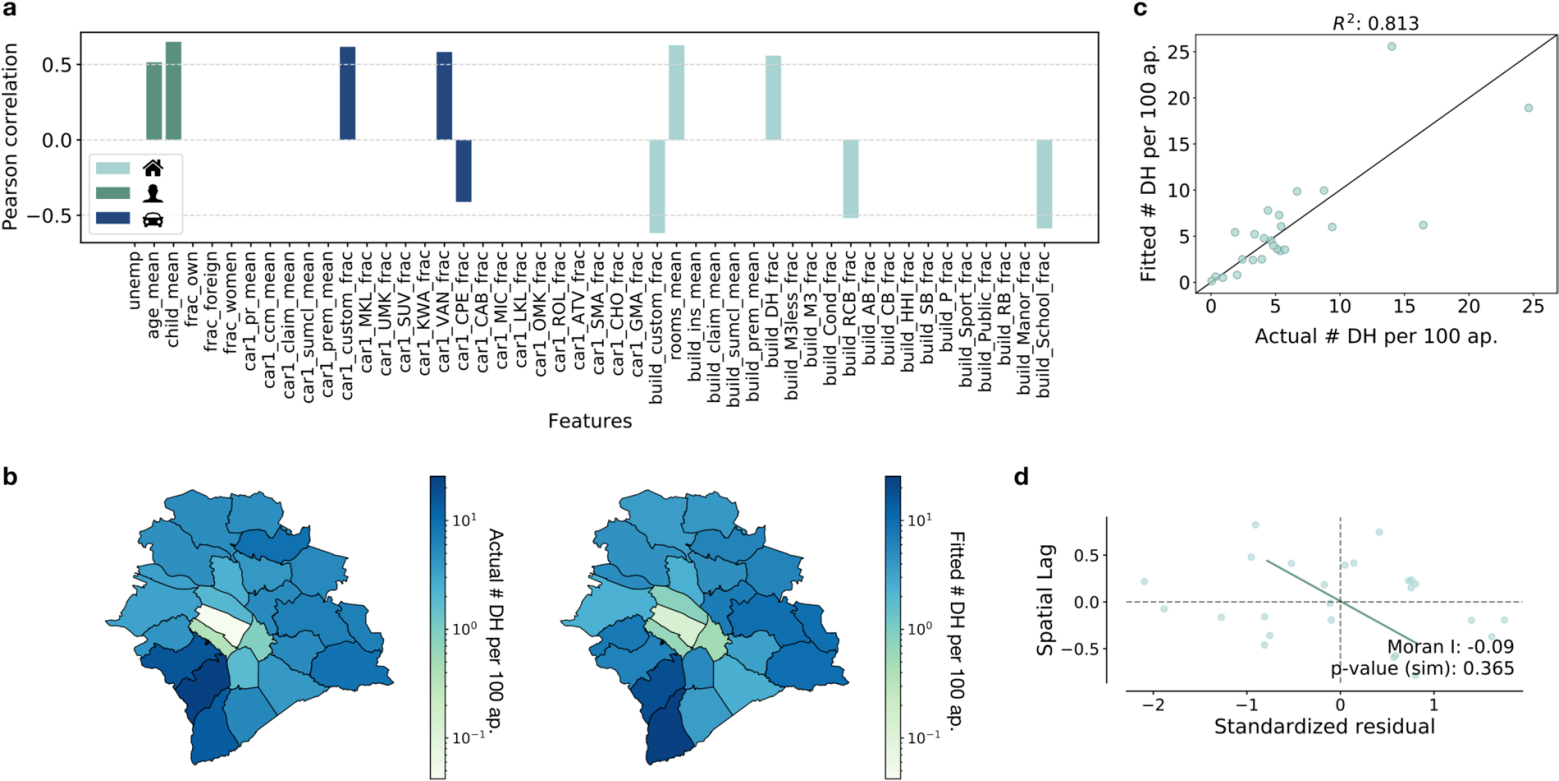
Prediction of geographical patterns at high-resolution: the case of Zurich. (**a**) shows the correlations between the number of detached houses per 100 apartments and all averaged features. The color scheme represents the domain of the feature (demographic, vehicle insurance and housing insurance). Only correlations that are statistically significant at 0.05 significance level are displayed. (**b**) maps the actual (left) and fitted (right) values of the number of detached houses per 100 apartments in 2019. (**c**) is a scatter plot of actual and fitted values of the number of detached houses per 100 apartments in 2019. (**d**) is a moran scatter plot of the standardized residuals. Queen weights were used. The data appears not to display any spatial correlation.

To build stronger evidence that our data can be used as proxies for demographic and socio-economic measures at high-geographical resolution, we investigate the ability of the insurance data to mimic geographical patterns observable within a single municipality. We focus on the municipality of Zurich, the largest municipality by number of postal areas (24) and use 2019 insurance data at ZIP-code level to model the number of detached houses per 100 apartments (again measured in 2019) (Fig. 9). We use a linear regression, with variables selection performed via Lasso. To account for the skewed distribution, we take a log-transformation of the target variable and center both inputs and target using z-scores. Despite the very limited sample size, the results of the analysis (Fig. 9(c)) show that the insurance data can be used to model geographical patterns at highly granular levels. The prediction appears to be more accurate for postal code areas associated to small values of the target variable - but overall the insurance data well reproduces the geographical heterogeneity in the measure, with areas in the south-west of Zurich being associated with the highest concentration of detached houses. Conversely, more central postal code areas are correctly predicted to have the lowest concentration of detached houses per 100 apartments.

## Usage Notes

### Data parsing

All files are provided in comma separated value format, with header on the first line and one record per line. This type of files is easily parsed with any programming language (e.g., R, Python) or spreadsheet utilities (e.g., OpenOffice Calc).

### Spatial definition

The spatial data at municipality level (combinedData_year.csv files) can be integrated with other datasets using the geographical area identifier–the first column in every file–as join key (BFS number). It is important to note that the boundaries of the geographical areas were periodically redefined in the past decades. For this reason, data at municipality level are only provided for 2,095 municipalities whose administrative boundaries have not changed in the last decades. The BFS nomenclature is available from the *swisstopo* (https://www.swisstopo.admin.ch/).

## Data Availability

The code used to validate our data is available at https://github.com/alibatti/LaMobiliereDatasetCode in the form of Python scripts.
